# Identification of MicroRNA–Potassium Channel Messenger RNA Interactions in the Brain of Rats With Post-traumatic Epilepsy

**DOI:** 10.3389/fnmol.2020.610090

**Published:** 2021-02-01

**Authors:** Zheng Li, Yixun Ma, Fengjuan Zhou, Xiao Jia, Jingjing Zhan, Huachao Tan, Xu Wang, Tiantong Yang, Quan Liu

**Affiliations:** ^1^Key Laboratory of Evidence Science, Institute of Evidence Law and Forensic Science, China University of Political Science and Law, Ministry of Education, Beijing, China; ^2^Collaborative Innovation Center of Judicial Civilization, Beijing, China; ^3^Hubei University of Police, Wuhan, China

**Keywords:** post-traumatic epilepsy, potassium channel, microRNA, RNA sequencing, gene annotation

## Abstract

**Background:** Dysregulated expression of microRNAs and potassium channels have been reported for their contributions to seizure onset. However, the microRNA–potassium channel gene interactions in traumatic brain injury-induced post-traumatic epilepsy (PTE) remain unknown.

**Methods:** PTE was induced in male rats by intracranial injection with ferrous chloride (0.1 mol/L, 1 μl/min) at the right frontal cortex. Electroencephalography was recorded at 60 min, as well as day 1, 7, and 30, and the behavioral seizures were assessed before injection and at different time points after injection. Rats were killed on day 30 after injection. The right frontal cortex samples were collected and subjected to high throughput messenger RNA (mRNA) and microRNA sequencing. A network of differentially expressed potassium channel mRNAs and microRNAs was constructed using OryCun2.0 and subjected to Gene Ontology and Kyoto Encyclopedia of Genes and Genomes analyses. The differential mRNA and microRNA expressions were verified using quantitative real-time-PCR. The microRNA–mRNA was subject to the Pearson correlation analysis.

**Results:** A PTE rat model was successfully established, as evidenced by behavioral seizures and epileptiform discharges on electroencephalography in PTE rats compared with sham rats. Among the 91 mRNAs and 40 microRNAs that were significantly differentially expressed in the PTE rat brain, 4 mRNAs and 10 microRNAs were associated with potassium channels. Except for potassium calcium-activated channel subfamily N member 2, the other three potassium channel mRNAs were negatively correlated with seven microRNAs. These microRNA–mRNA pairs were enriched in annotations and pathways related to neuronal ion channels and neuroinflammation. Quantitative real-time-PCR and correlation analysis verified negative correlations in miR-449a-5p-KCNH2, miR-98-5p-KCNH2, miR-98-5p-KCNK15, miR-19b-3p-KCNK15, and miR-301a-3p-KCNK15 pairs.

**Conclusion:** We identified microRNA–potassium channel mRNA interactions associated with PTE, providing potential diagnostic markers and therapeutic targets for PTE.

## Introduction

Post-traumatic epilepsy (PTE) is a recurrent seizure disorder secondary to traumatic brain injury (TBI), accounting for 20% of acquired epilepsy and 5% of all epilepsy (Frey, 2003). The incidence of PTE in TBI patients within 9 years is more than twice of that in non-TBI patients and is positively correlated with the severity of TBI (DeGrauw et al., 2018). The TBI patients who develop seizure symptoms at an early stage have a significantly increased risk of PTE compared with those without seizure symptoms. Seizure episodes are closely associated with excessive discharges in the neurons (Chang and Lowenstein, 2003) resulting from the increased neuronal excitability after TBI (D'Ambrosio et al., 1999); however, the precise molecular mechanisms underlying PTE remains largely unknown.

Potassium channels play critical roles in neuronal excitability and plasticity (Misonou, 2010) by controlling ion flow across membranes, generating action potentials, and maintaining neuronal homeostasis (Kuang et al., 2015). Over a hundred different potassium channel subtypes have been identified so far, and many of them are widely expressed in the mammalian brain and are linked with epilepsy (Wei et al., 2017). Unlike genetic epilepsy, PTE is acquired secondary to TBI, and no pathogenic genetic alterations have been identified for PTE development (SoRelle et al., 2019). Although evidence has suggested that genetic factors (Diamond et al., 2015a,b; Cotter et al., 2017), along with DNA methylation (Debski et al., 2016), might contribute to PTE development, these factors do not have long-term impacts. TBI-induced mRNA alterations might drive the occurrence of PTE (Drexel et al., 2015; Lipponen et al., 2018). For example, the transcription of potassium voltage-gated channel subfamily D member 2 encoding A-type potassium channel subunit Kv4.2 is downregulated after TBI, which is associated with hyperexcitability in the post-traumatic rat hippocampus and contributes to subsequent epilepsy (Lei et al., 2012). On the other hand, the transcription of KCNQ2 encoding M-type potassium channel Kv7.2 is upregulated after TBI, reducing TBI-induced spontaneous seizures (Vigil et al., 2020). However, these findings have not been verified in PTE models.

The evidence of PTE-related alterations in potassium channel gene transcription also attracts great attention to microRNA (miRNA)-mediated regulation on potassium channel genes. MicroRNAs are small non-coding RNAs (20–25 nucleotides in length) generated by the cleavage of primary transcripts, playing important roles in multiple physiological and pathological processes, including development, virus infection, metabolism, and cell fate. MicroRNA recognizes their mRNA targets through base pairing, leading to mRNA degradation or translational repression (Bernstein et al., 2001). Studies have shown that many miRNAs are associated with seizure episodes, including miR-134, miR-181a, miR-146a, miR-124, miR-199a, miR-128, miR-155, miR-211, let-7b-3b, and let-7b-3p (Bekenstein et al., 2017; Ma, 2018; Mills et al., 2020). However, to the best of our knowledge, only miR-23a and miR-451 are dysregulated in PTE rat models (Kamnaksh et al., 2019), and the miRNA–mRNA interactions in PTE remain unidentified. Considering that miRNAs function through mRNAs (Abu-Halima et al., 2018; Zhang et al., 2019) and that potassium channels play critical roles in PTE development, it is necessary to reveal miRNA–potassium channel mRNA interactions for a better understanding of the pathogenesis of PTE.

In this study, to reveal miRNA–potassium channel mRNA interactions involved in the development of PTE, we established a ferrous chloride (FeCl_2_)-induced PTE rat model, identified differentially expressed miRNAs and potassium channel mRNAs in PTE rat brain using RNA sequencing, constructed a miRNA–mRNA network using bioinformatics approaches, and validated the negative correlations between the candidate miRNAs and mRNAs. Our results suggest that miRNA–potassium channel mRNA interactions are involved in the development of PTE, serving as potential diagnostic biomarkers and therapeutic targets for PTE.

## Materials and Methods

### Ethics Statement

The Animal Research Ethics Committee of the Institute of Evidence Science, China University of Political Science and Law, approved the animal study (#2019012). All the animal experiments and procedures were performed following the guidelines of the Weatherall Committee and the National Centre for the Replacement, Refinement, and Reduction of Animals in Research (NC3Rs; London, UK).

### Animals and Sample Preparation

A total of 15 male Sprague-Dawley rats (7–8-months old, weighing 210–230 g) were purchased from Beijing Laboratory Animal Research Center (Beijing, China) and housed in a 12-h light/dark cycle with free access to water and food. After a 72-h acclimatization period, the rats were randomly divided into sham and PTE groups (*n* = 6/group). A FeCl_2_-induced PTE model was established as previously described (Ueda et al., 1998). Briefly, the rats were anesthetized with 2% pentobarbital sodium (40 mg/kg) *via* intraperitoneal injection and placed in a stereotactic device (Nanjing Medease Science and Technology, Jiangsu, China). The electrodes were fixed to the rat frontal and occipital lobes and connected with a biosignal acquisition system (Shanghai creaform3d information, Shanghai, China) for EEG collection. The rats in the PTE group were injected with 10-μl FeCl_2_ (0.4 mol/L, 1 μl/min) at the right frontal cortex (2.0 mm anterior to bregma, 3.0 mm from midline, and 2.0 mm depth) using a microinjector. The sham group underwent the same procedures except for the injection. The rats were killed on day 30 after the operation. The brain tissue samples surrounding the injection sites were collected and stored at −80°C for further experiments.

### Behavioral Assessment and Electroencephalography Monitoring

The severity of seizures was assessed at 1 h before the operation, 1 h after operation, and once daily thereafter for 30 consecutive days according to the Racine's scale (Racine, 1972): 0, no abnormality; 1, staring; 2, head nodding or wet-dog shakes, with or without facial twitching; 3, unilateral forelimb clonus; 4, bilateral forelimb clonus and continuous head nodding; 5, exacerbated bilateral forelimb clonus, loss of balance and falling, or generalized tonic–clonic seizures.

A 1-h EEG, including the first 15 min and the blocks of 10 min at 5-min intervals, was recorded at 1 h before the operation and at 1 h and 1, 7, and 30 days after the operation.

The criteria of a successful PTE model include: (1) Racine's score >4, (2) sharp waves or spike waves on EEG, and (3) paroxysmal or continuous abnormal discharges on EEG.

### Total RNA Isolation, Library Construction, and Sequencing

Total RNA was isolated from frontal lobe brain tissues adjacent to the FeCl_2_ injection region of rats using TRIzol™ Reagent (#15596026, Thermo Fisher Scientific, Waltham, MA, USA) following the manufacturer's protocol. The RNA quality was monitored on 1% agarose gels. The purity and quantity of RNA were determined using a NanoPhotometer® device (Implen, Camarillo, CA, USA) and a Qubit® 2.0 kit, respectively. The RNA integrity was examined using an RNA Nano 6000 kit on a Bioanalyzer 2100 system (Life Technologies, Carlsbad, CA, USA).

For each sample, a total of 3-μg RNA was loaded to generate the sequencing library using a NEBNext® Ultra™ RNA library prep kit for Illumina® (NEB, USA) following the manufacturer's instructions. The RNA was purified using an AMPure XP system, and the RNA quality was evaluated on an Agilent Bioanalyzer 2100 system. Gene clustering was performed using the TruSeq PE Cluster Kit v3-cBot-HS (Illumina, San Diego, CA, USA). The 150-bp paired-end reads were generated for mRNA sequencing, and the 50-bp single-end reads were generated for miRNA sequencing, using an Illumina HiSeq 2500 sequencer.

### RNA Sequencing Data Analysis

Clean reads were obtained from raw reads (FASTQ format) by removing adapter- or ploy-N-containing reads and low-quality reads. Q20, Q30, and GC contents were calculated. Clean reads were aligned with the reference genome (rat release-91) downloaded from the Ensembl database using Bowtie v2.2.3 (Ghosh and Chan, 2016) and TopHat v2.0.12 (Trapnell et al., 2012). The fragments per kilobase of transcript per million mapped reads of each transcript was calculated using (Trapnell et al., 2010) Cuffdiff v2.1 (Ghosh and Chan, 2016). MicroRNA expression was compared with the expression of miRNA precursors and corresponding mature miRNAs in miRBase v22 (Kozomara and Griffiths-Jones, 2014) using miRDeep2 (Friedländer et al., 2012). The differentially expressed mRNA was analyzed using Cuffdiff v2.1. The differentially expressed miRNA was analyzed using the DESeq R package. Genes with an adjusted *P*-value < 0.05 were identified as differentially expressed genes. The miRNA–mRNA interactions were predicted using RNAhybrid, PITA, and miRanda (John et al., 2004). The target genes of differentially expressed miRNAs were subjected to Gene Ontology (GO) and Kyoto Encyclopedia of Genes and Genomes (KEGG) analysis using GOseq (Young et al., 2010) and KEGG Orthology Based Annotation System (Mao et al., 2005).

### Quantitative Real-Time PCR

Quantitative real-time (qRT)-PCR was conducted to measure the expression of differentially expressed miRNAs and mRNAs. For mRNA expression determination, complementary DNA was synthesized using SuperScript™III SuperMix (Thermo Fisher Scientific, Waltham, MA, USA) according to the manufacturer's instructions. Primers of KCNH2, KCNK15, and Slc24a4 were designed using National Center for Biotechnology Information primer-blast (http://www.ncbi.nlm.nih.gov/tools/primer-blast/), and the sequences are summarized in Table 1. Glyceraldehyde 3-phosphate dehydrogenase was used as an internal control. PCR was performed using SYBR Power Plus Master Mix (Thermo Fisher Scientific). Conditions for real-time PCR were 95°C for 10 min, followed by 40 cycles of 95°C for 15 s, 60°C for 60 s. Melting curve analysis was performed from 65 to 95°C with increments of 0.5°C. For miRNA expression determination, complementary DNA was synthesized using a TaqMan®MiRNA reverse transcription kit (Applied Biosystems, Foster City, CA, USA) following the manufacturer's protocol. PCR was performed using TaqMan®MiRNA Assays and TaqMan®Universal PCR master mix (Applied Biosystems) on an Applied 7500 device. Conditions for real-time PCR were 95°C for 10 min, followed by 50 cycles of 95°C for 15 s, 60°C for 60 s. Melting curve analysis was performed from 65 to 95°C with increments of 0.5°C. The miRNA primers are summarized in Table 2. U6 small nuclear RNA was used as an internal control. The mRNA or miRNA expression was quantified using the 2–ΔΔCt method.

**Table 1:** Primers for quantitative real-time PCR.

**Primer name**	**Primer sequence**
GAPDH1qPCRF	5′-CACCAGCATCACCCCATT-3′
GAPDH1qPCRR	5′-CCATCAAGGACCCCTTCATT-3′
Kcnk15qPCRF	5′-GGTGGTCCTGCGATTCCTTG-3′
Kcnk15qPCRR	5′-GCACTGCTTCTGGGCTCAAA-3′
Kcnh2qPCRF	5′-CAGTTCGTGGCGTTTGAGGAG-3′
Kcnh2qPCRR	5′-GCCTGGATCTGAGCCATGTC-3′

**Table 2:** MicroRNA primers for quantitative real-time PCR.

**MicroRNA**	**Primer sequence**
miR-30e-5p	5′-CTTCCAGTCAAGGATGTTTACA-3′
miR-98-5p	5′-AACAATACAACTTACTACCTCA-3′
miR-19b-3p	5′-TCAGTTTTGCATGGATTTGCACA-3′
miR-138-5p	5′-CGGCCTGATTCACAACACCAGCT-3′
miR-301a-3p	5′-GCTTTGACAATACTATTGCAC-3′
miR-449a-5p	5′-ACCAGCTAACAATACACTGCCA-3′
miR-139-3p	5′-CTCCAACAGGGCCGCGTCTCCA-3′

### Statistical Analysis

Data are expressed as mean ± standard deviation. Statistical analysis was performed using SPSS 25.0 (IBM, Armonk, NY, USA). Graphs were generated using GraphPad Prism 8 (San Diego, CA, USA). The correlation between mRNA and miRNA was assessed using Pearson's correlation coefficient. Different groups were compared using a one-way analysis of variance, followed by a least significant difference *t*-test. A *P*-value < 0.05 was considered statistically significant.

## Results

### Successful Establishment of a Post-traumatic Epilepsy Rat Model

To evaluate whether the PTE rat model was successfully established, we examined behavioral seizures and EEG in the rats before and after FeCl_2_ injection. We found that compared with the sham rats, the rats in the PTE group developed behavioral seizures after FeCl_2_ injection, including paroxysmal binocular immobility, staring, head nodding or wet-dog shakes, facial twitching, and generalized tonic–clonic seizures. The behavioral seizures frequently occurred within 3 days after injection and were declined thereafter until regular behavioral seizures developed at around 15 days after injection. The total Racine's scores of the PTE group was 810, with a mean value >4 for each rat.

**Figure 1:**
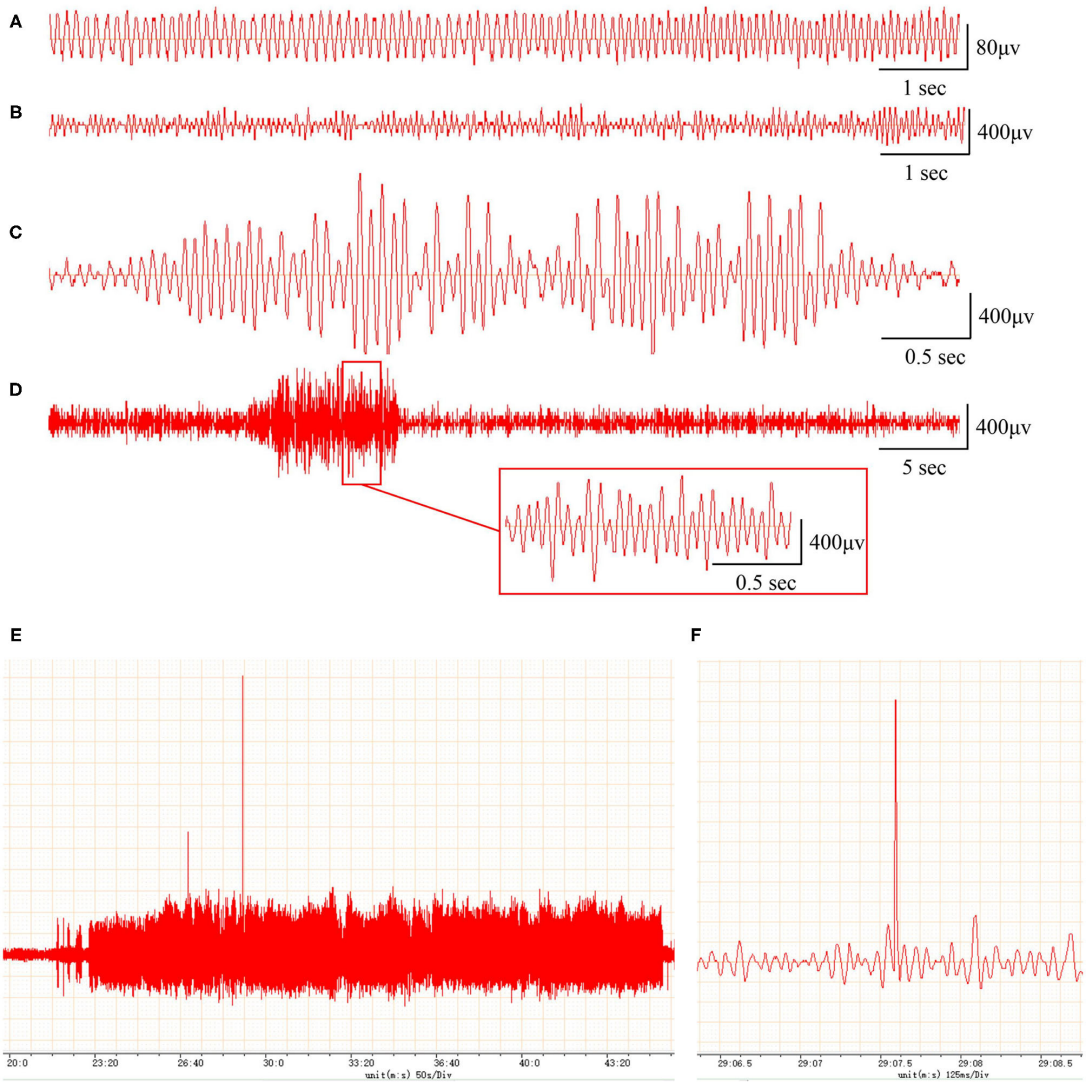
Rats with FeCl_2_ injection-induced post-traumatic epilepsy (PTE) showed epileptiform discharges on electroencephalogram (EEG). A total of 12 rats were randomly divided into sham and PTE groups (*n* = 6/group). Rats in PTE group were injected with 10-μl FeCl_2_ (0.4 mol/L, 1 μl/min) at right frontal cortex. Sham group underwent same procedures except for injection. A 1-h EEG, including first 15 min and blocks of 10 min at 5-min intervals, was recorded at 1 h before injection and at 1 h and 1, 7, and 30 days after injection. Representative EEGs are shown. **(A)** Normal EEG at 60 min before injection. Multiple spikes **(B)**, two- and three-phase sharp waves **(C)**, 10-s continuous abnormal discharges **(D)**, 20-min explosive abnormal discharges with occasional high-amplitude spikes **(E)**, and a sudden high-amplitude spike **(F)** were observed on day 30 after injection.

In addition, compared with the EEG of the sham rats (frequency 5–10 Hz, amplitude <200 μV; Figure 1A), the EEG of the PTE rats exhibited multiple epileptiform discharges, such as sharp-wave polyspikes (Figure 1B), waves (Figure 1C), spikes (Figures 1E,F), and continuous abnormal discharges (Figure 1D) with a maximum amplitude of 1,000 μV. These results indicate that FeCl_2_ injection successfully induces PTE in rats.

### Differentially Expressed Messenger RNAs in Frontal Lobes of Post-traumatic Epilepsy Rats

**Figure 2:**
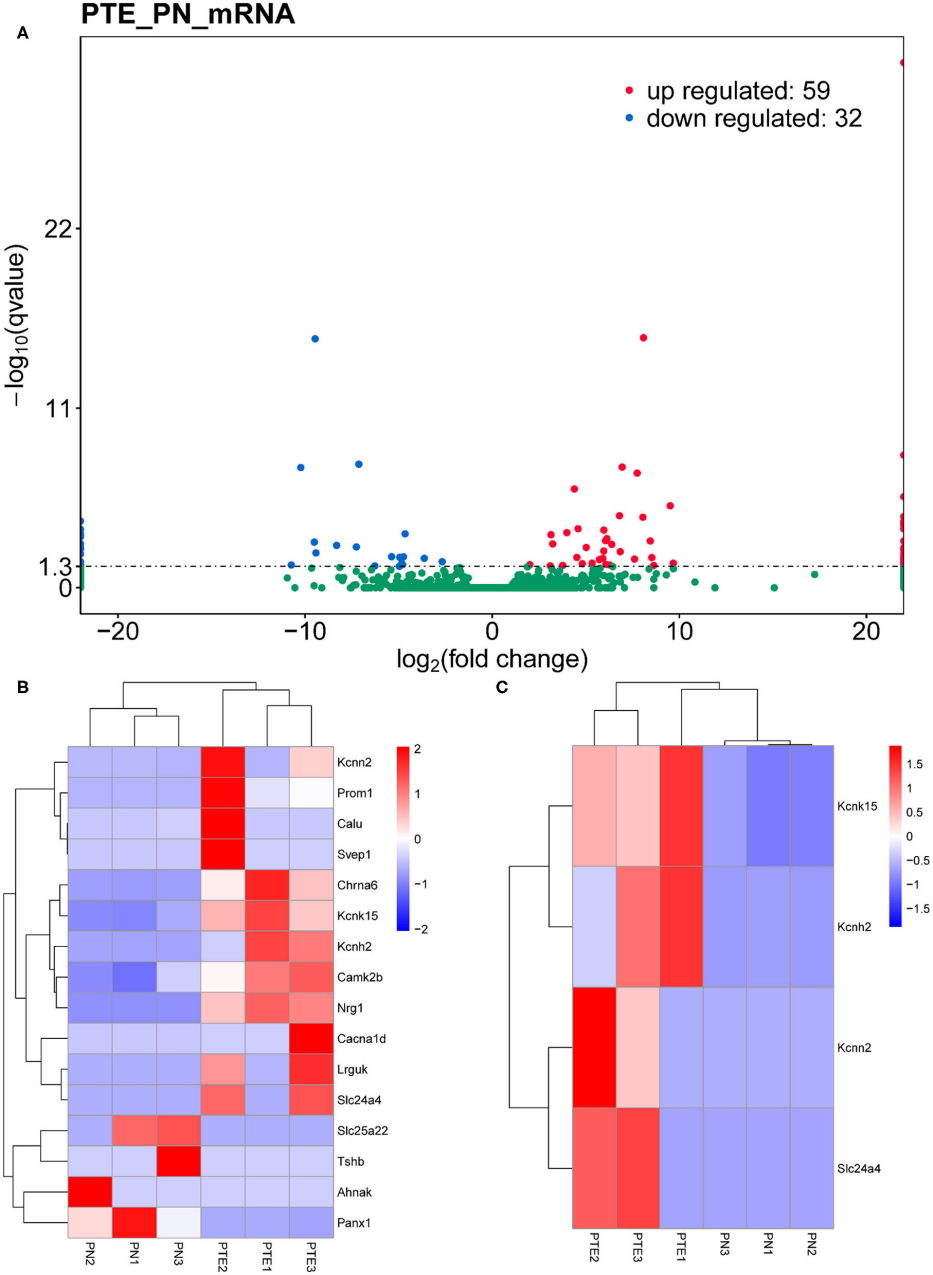
mRNA transcriptome analysis in rat frontal lobe by RNA sequencing (RNA-Seq). **(A)** A volcano plot of RNA-seq transcriptome data displays gene expression values of PTE rats compared with those of sham rats. Significantly differentially expressed genes (adjusted *P*-value <0.05) are highlighted in red (upregulated) or blue (downregulated). Non-differentially expressed genes are highlighted in green. *n* = 6. **(B)** Heat map of 12 significantly upregulated and four significantly downregulated ion channel-related genes in PTE rats compared with sham rats. **(C)** Heat map of four significantly upregulated potassium channel-encoding genes in PTE rats compared with sham rats. Adjusted *P*-value <0.05; *n* = 6.

To identify differentially expressed mRNAs involved in PTE, we performed high-throughput RNA sequencing using the brain samples from rats. As shown in Figure 2A, compared with sham rats, PTE rats had 91 significantly differentially expressed mRNAs (59 upregulated and 32 downregulated) in the frontal lobes. GO annotation analysis revealed that among these differentially expressed mRNAs, 16 mRNAs (12 upregulated and four downregulated) were associated with ion channels (Figure 2B, Table 3). Among the 12 upregulated ion channel-related genes, KCNH2, KCNK15, Slc24a4, and KCNN2 encode potassium channel proteins (Figure 2C, Table 3).

**Table 3:** Gene annotation of differentially expressed ion channel-related genes in PTE rats.

**gene_name**	**PTE_FPKM**	**PN_FPKM**	**log2 (foldchange)**	***p*-Value**	***q* Value**	**Gene_description**
Kcnn2	2.477944667	0.00669	8.532922046	3.76E-05	0.014185449	“Potassium channel domain∥Calmodulin-binding domain∥Potassium channel, calcium-activated, SK”
Prom1	3.479028333	0	Inf	1.08E-09	2.65E-06	Prominin
Calu	5.7256	0.006981	9.679777458	0.000111681	3.15E-02	“Calumenin∥EF-hand domain pair∥EF-hand domain∥EF-Hand 1, calcium-binding site”
Svep1	0.236109	0	Inf	5.41E-05	0.018150161	“Tyrosine-protein kinase ephrin type A/B receptor-like∥EGF-like, conserved site∥Sushi/SCR/CCP domain∥EGF-type aspartate/asparagine hydroxylation site∥Concanavalin A-like lectin/glucanase domain∥EGF-like domain∥EGF-like calcium-binding domain∥Growth factor receptor cysteine-rich domain∥EGF-like calcium-binding, conserved site∥Pentaxin-related∥von Willebrand factor, type A∥Green fluorescent protein-like∥HYR domain”
Chrna6	0.993328333	0.015922333	5.963146987	1.17E-05	0.005575596	“Neurotransmitter-gated ion-channel ligand-binding domain∥Neurotransmitter-gated ion-channel, conserved site∥Nicotinic acetylcholine receptor∥Nicotinic acetylcholine-gated receptor, transmembrane domain∥Neurotransmitter-gated ion-channel∥Neurotransmitter-gated ion-channel transmembrane domain”
Kcnk15	2.198411333	0.095449	4.525587454	3.62E-05	0.013831119	“Two pore domain potassium channel∥Two pore domain potassium channel, TASK family∥Potassium channel domain∥Potassium channel subfamily K member 15”
Kcnh2	2.334764333	0.003191333	9.514901914	4.44E-09	9.52E-06	“Potassium channel, voltage-dependent, EAG/ELK/ERG∥PAS domain∥RmlC-like jelly roll fold∥Potassium channel, voltage-dependent, ERG∥PAC motif∥Cyclic nucleotide-binding domain∥Ion transport domain∥Cyclic nucleotide-binding-like∥PAS-associated, C-terminal”
Camk2b	4.608144	0.536467	3.102624467	0.000203298	0.04531143	“Calcium/calmodulin-dependent/calcium-dependent protein kinase∥Calcium/calmodulin-dependent protein kinase II, association-domain∥Protein kinase, ATP binding site∥NTF2-like domain∥Protein kinase-like domain∥Protein kinase domain∥Serine/threonine-protein kinase, active site”
Nrg1	0.534145667	0	Inf	5.36285E-08	8.10241E-05	“Immunoglobulin I-set∥Neuregulin, C-terminal∥Immunoglobulin subtype 2∥Immunoglobulin-like domain∥Immunoglobulin-like fold∥Immunoglobulin subtype∥Neuregulin-1∥EGF-like, conserved site∥EGF-like domain”
Cacna1d	0.643110333	0	Inf	3.40E-05	0.013308316	“Voltage-gated calcium channel subunit alpha, C-terminal∥Voltage-dependent channel, four helix bundle domain∥Voltage-dependent L-type calcium channel, IQ-associated domain∥Voltage-dependent calcium channel, alpha-1 subunit∥Voltage-dependent calcium channel, alpha-1 subunit, IQ domain∥Ion transport domain∥Voltage-dependent calcium channel, L-type, alpha-1 subunit∥Voltage-dependent calcium channel, L-type, alpha-1D subunit”
Lrguk	0.561985667	0	Inf	2.14E-05	0.008819948	“Leucine-rich repeat domain, L domain-like∥P-loop containing nucleoside triphosphate hydrolase∥Guanylate kinase/L-type calcium channel beta subunit∥Guanylate kinase-like domain∥Leucine-rich repeat, typical subtype∥Leucine-rich repeat”
Slc24a4	0.544000667	0	Inf	0.000205718	0.045344842	Sodium/potassium/calcium exchanger∥Sodium/potassium/calcium exchanger 4∥Sodium/calcium exchanger membrane region
Slc25a22	0	5.435152333	#NAME?	2.98E-06	0.001981222	Mitochondrial carrier domain∥Mitochondrial carrier protein∥Mitochondrial substrate/solute carrier
Tshb	0	27.077657	#NAME?	0.00020395	0.04531143	“Cystine-knot cytokine∥Glycoprotein hormone subunit beta, cystine knot∥Gonadotropin, beta subunit, conserved site∥Gonadotropin, beta subunit”
Ahnak	0	2.561203	#NAME?	8.02E-05	0.024947173	PDZ domain
Panx1	0.002705	0.859885	−8.312371324	4.16E-06	0.002535909	-∥Innexin

### Differentially Expressed MicroRNAs in Frontal Lobes of Post-traumatic Epilepsy Rats

**Figure 3:**
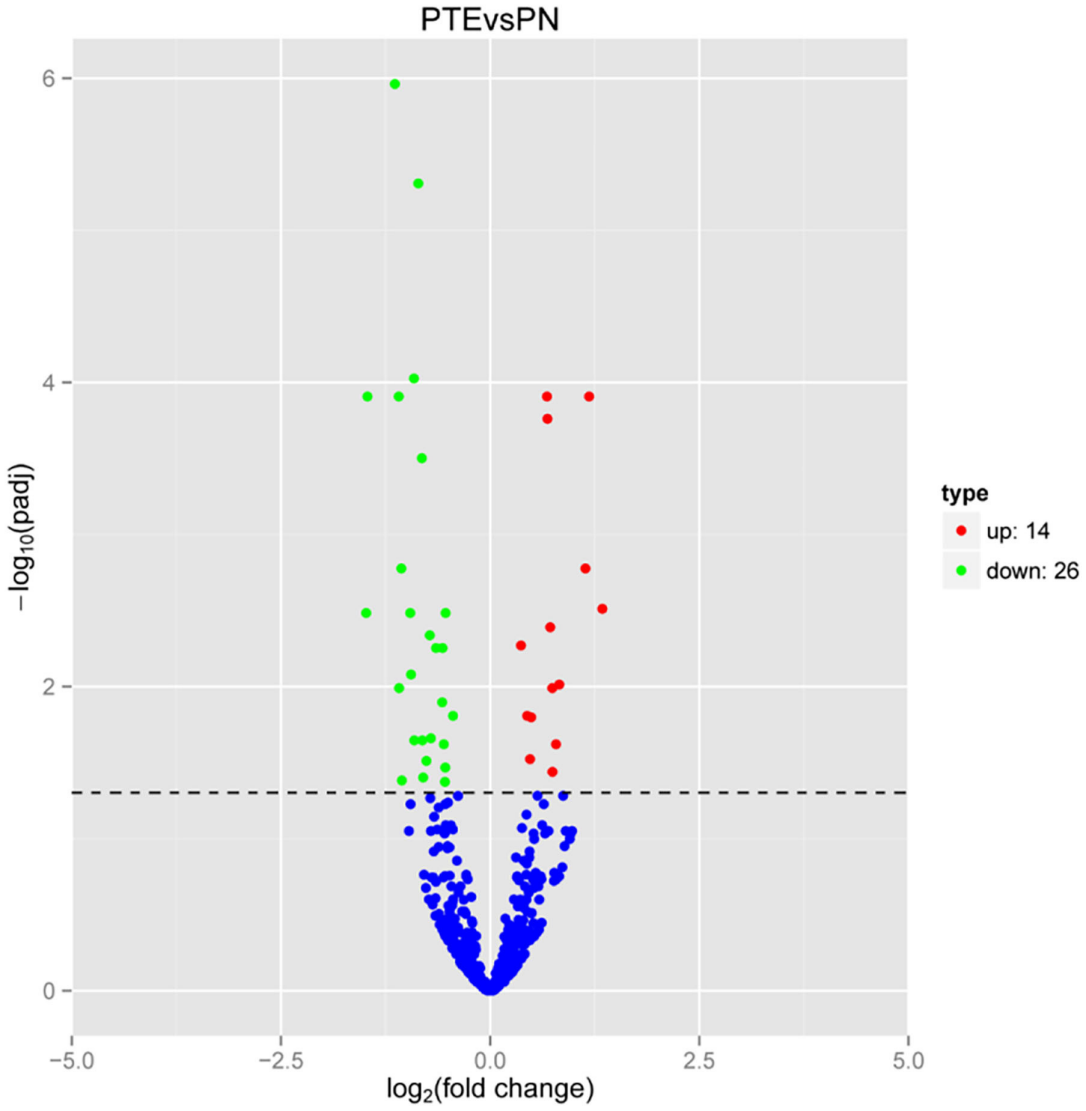
MicroRNA transcriptome analysis in rat frontal lobe by RNA-Seq. A volcano plot of RNA-seq microRNA transcriptome data displays miRNA expression values of PTE rats compared with those of sham rats. Significantly differentially expressed microRNAs (adjusted *P*-value < 0.05) are highlighted in red (upregulated) or green (downregulated). Non-differentially expressed microRNAs are highlighted in blue. *n* = 6.

Considering the interaction between mRNAs and miRNAs, we performed high-throughput sequencing to identify differentially expressed miRNAs in PTE rats. As shown in Figure 3, compared with sham rats, PTE rats had 40 significantly differentially expressed miRNAs (14 upregulated and 26 downregulated) in the frontal lobes.

### Construction of MicroRNA–Messenger RNA Regulatory Network

**Figure 4:**
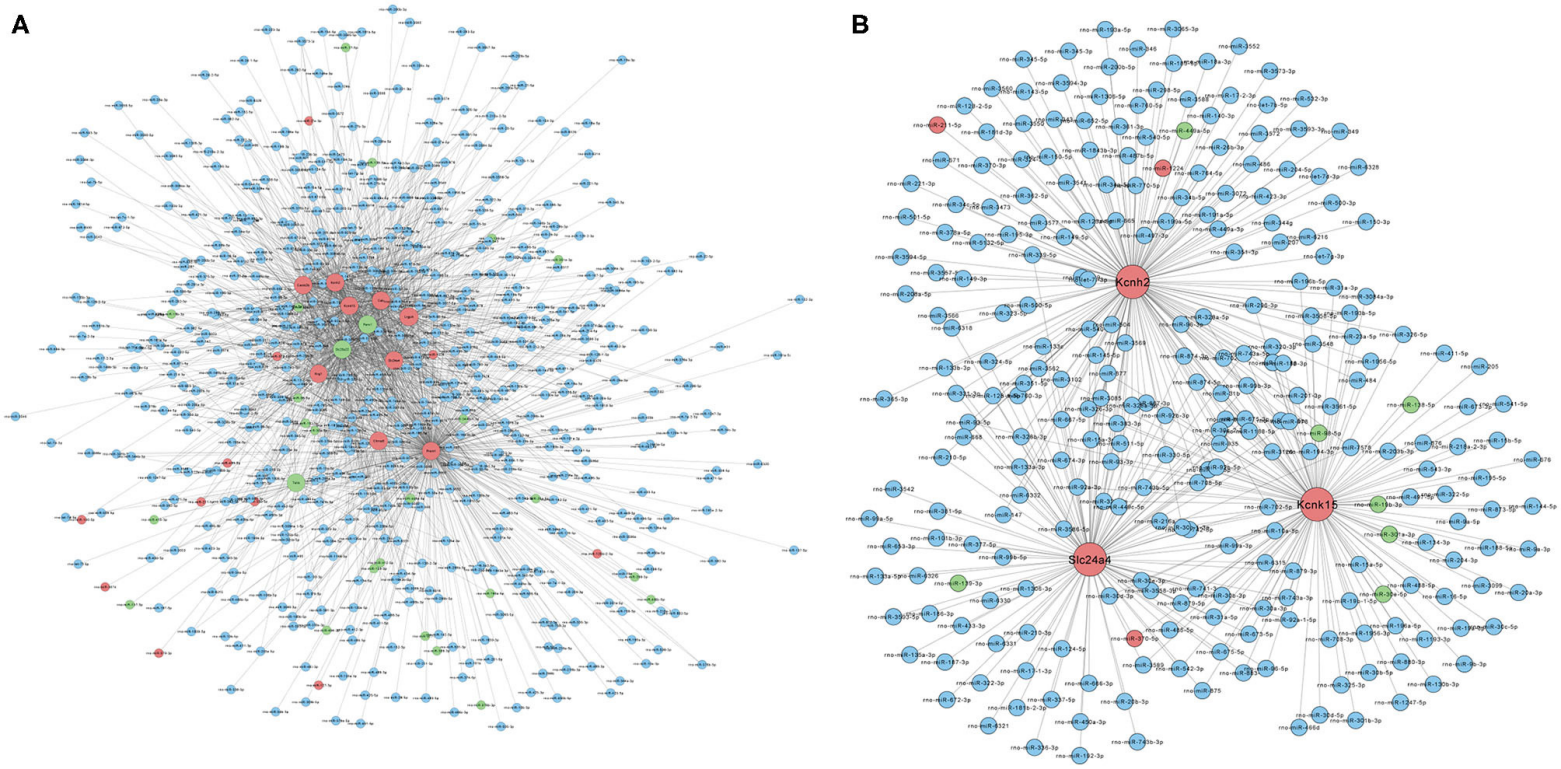
Prediction of microRNA–mRNA interaction. **(A)** Interaction network of 1,765 microRNAs with 12 ion channel-related mRNAs. **(B)** Interaction network of 396 microRNAs with three potassium ion channel-encoding mRNAs. Larger dots indicate mRNAs. Smaller dots indicated microRNAs. Red indicates upregulated mRNAs or microRNAs. Green indicated downregulated mRNAs or microRNAs. Blue indicates unchanged mRNAs or microRNAs.

Based on the RNA-seq results, we further analyzed the interactions between differentially expressed mRNAs and miRNAs by constructing a miRNA–mRNA network. Figure 4A shows the regulatory network of 1,765 miRNAs and 12 ion channel-related mRNAs, excluding KCNN2 and Aknak without matched miRNAs as well as Svep1 and Cacna1d, whose targeting miRNAs were not differentially expressed in PTE rats. Figure 4B shows the regulatory network of 396 miRNAs and three potassium channel mRNAs, excluding KCNN2 without matched miRNAs. Among the 396 miRNAs, 7 miRNAs negatively correlated with corresponding mRNAs, including miR-98-5p and miR-449a-5p, which negatively correlated with KCNH2; miR-138-5p, miR-19b-3p, miR-301a-3p, miR-30e-5p, and miR-98-5p, which negatively correlated with KCNK15; and miR-139-3p, which negatively correlated with Slc24a. In addition, three miRNAs positively correlated with corresponding mRNAs, including miR-211-5p and miR-1224, which positively correlated with KCNH2 and miR-370-5p, which positively correlated with Slc24a4.

### Characterization of Potassium Channel-Associated MicroRNAs by Gene Ontology Annotation and Kyoto Encyclopedia of Genes and Genomes Pathway Enrichment Analysis

**Figure 5:**
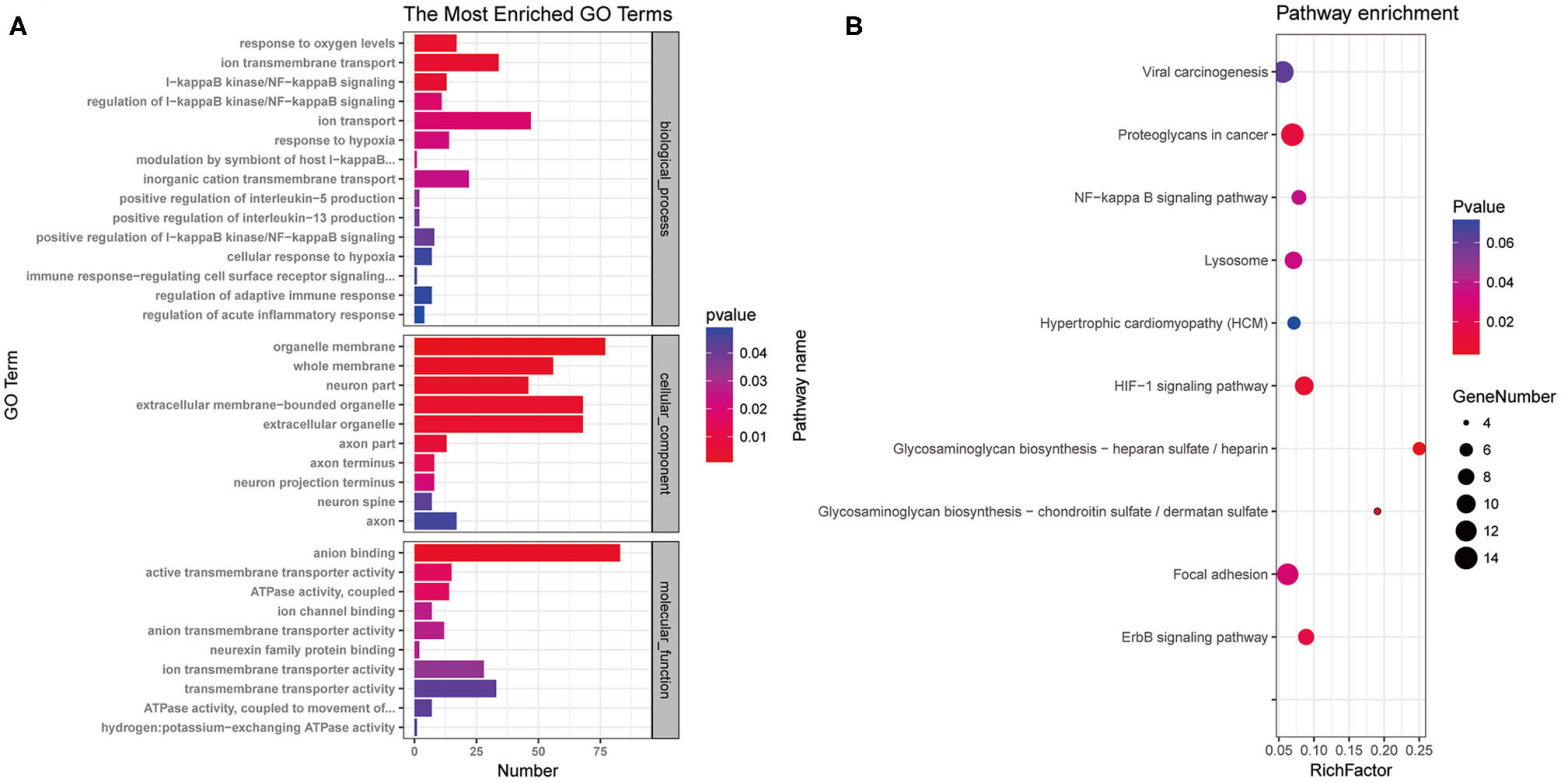
Gene Ontology annotation and Kyoto Encyclopedia of Genes and Genomes pathway analysis of differentially expressed microRNAs associated with potassium channels. **(A)** miRNAs were classified into three functional categories. Fifteen biological process annotations, 10 cellular component annotations, and 10 molecular function annotations were shown *P* < 0.05. **(B)** Top 10 Kyoto Encyclopedia of Genes and Genomes pathways *P* < 0.05.

To characterize the functions of the potassium channel-associated differentially expressed miRNAs, we conducted GO and KEGG enrichment analyses. As shown in Figure 5A, the 396 miRNAs were classified into three functional categories (biological process, cellular component, and molecular function). The miRNAs were enriched in biological process annotations, such as response to oxygen level, ion transmembrane transport, I-kappa B kinase/nuclear factor (NF)-kappa B signaling, and regulation of acute inflammatory response; cellular component annotations, such as whole membrane, neuron part, and axon part; and molecular function annotations, such as anion binding, ion transmembrane transporter activity, and neurexin family protein binding. KEGG analysis revealed that these miRNAs were associated with the “HIF-1signaling pathway,” “ErbB signaling pathway,” and “NF-kappa B signaling pathway” (Figure 5B). These data suggest that the potassium channel-related miRNAs and their target mRNAs play important roles in regulating neuronal ion channels and neuroinflammation.

### Evaluation of Correlation Between MicroRNAs and Potassium Channel Messenger RNAs

**Figure 6:**
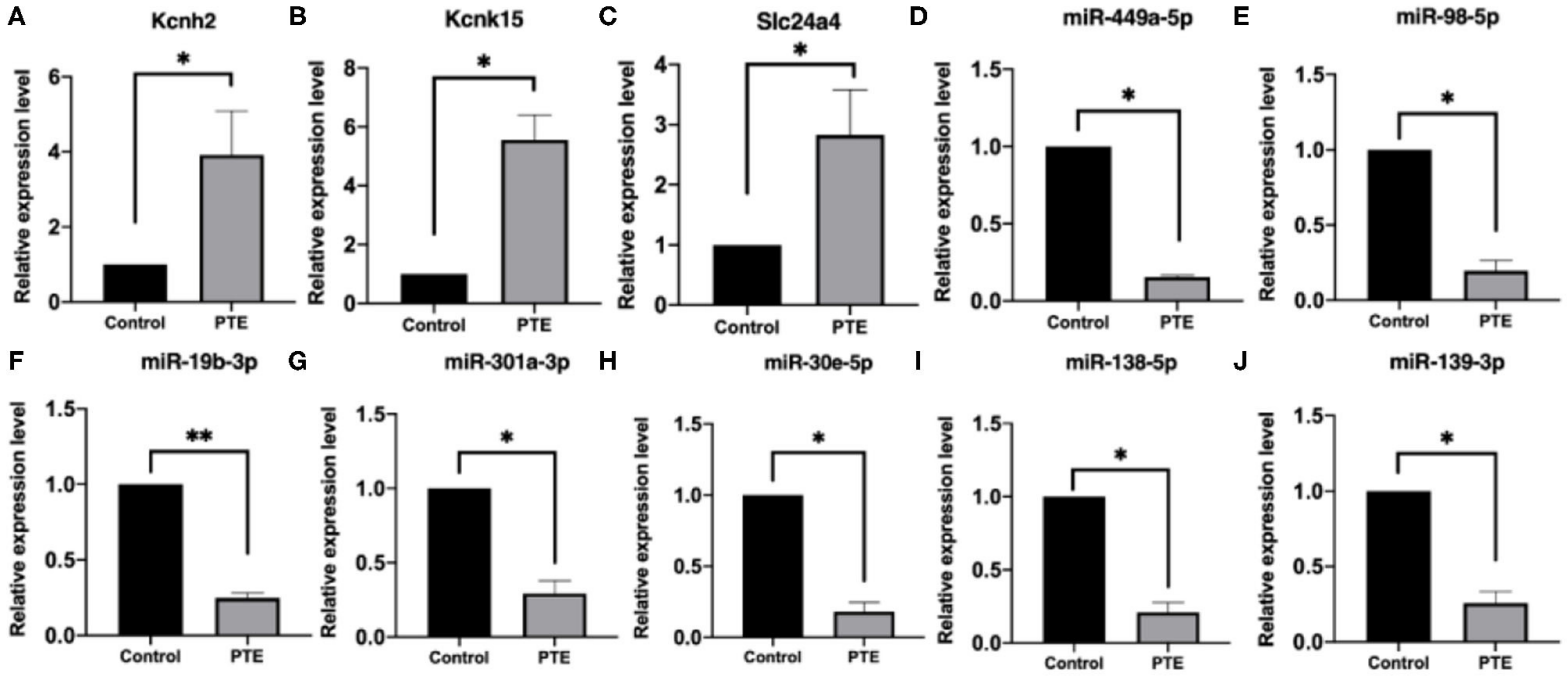
Quantitative real-time PCR verification of differentially expressed potassium channel mRNAs and microRNAs. *P < 0.05, **P < 0.01 vs. sham group. **(A–C)** The expression of verified mRNAs (KCNH2, KCNK15, and SLC24A4) in PTE group was significantly increased compared with the control group. **(D–J)** The expression of verified microRNAs (miR-449a-5p,miR-98-5p,miR-19b-3p, miR301A-3p, miR-30E-5p,miR-138-5p, and miR-139-3p) in the PTE group was significantly decreased compared with the control group.

To verify the alterations in the expression of candidate miRNA–potassium channel mRNA pairs, we performed qRT-PCR to measure their levels in rat brain samples. As shown in Figure 6, consistent with that observed in high-throughput analysis, KCNH2, KCNK15, and Slc24a4 mRNA levels in PTE rats were remarkably increased, whereas miR-138-5p, miR-19b-3p, miR-301a-3p, miR-30e-5p, miR-98-5p, miR-449a-5p, and miR-139-3p levels in PTE rats were markedly decreased (all *P* < 0.05), compared with those in sham rats.

**Figure 7:**
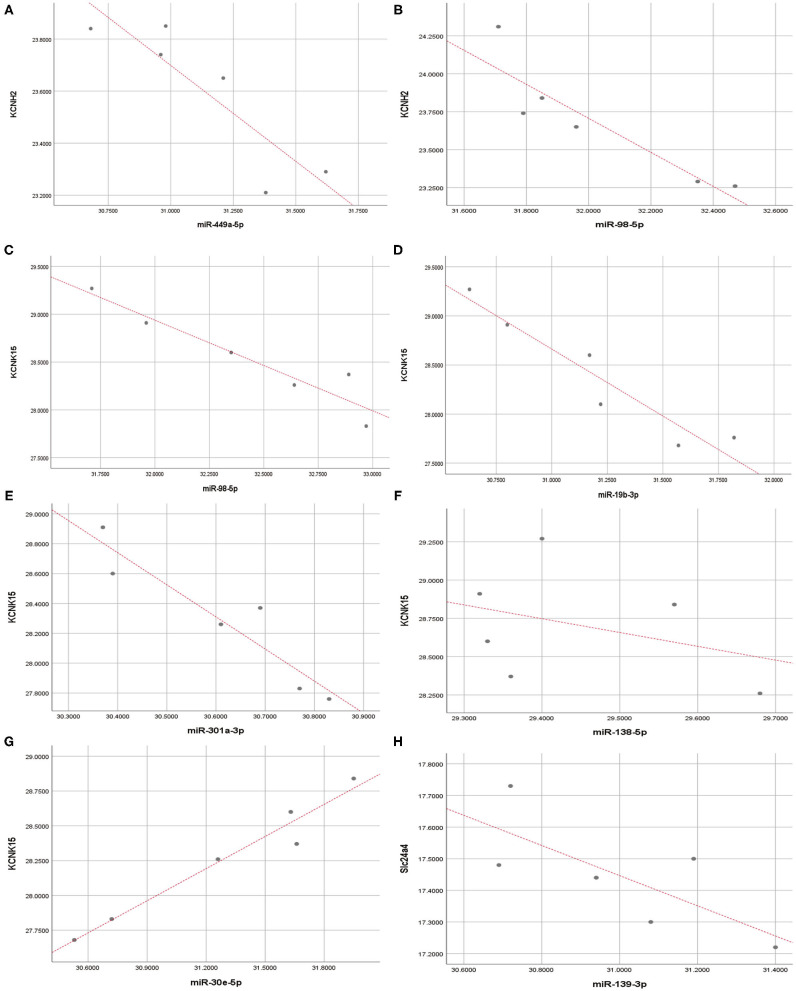
Correlation analysis. Pearson correlation analysis was conducted to evaluate the correlation between microRNAs and potassium channel mRNAs. **(A,B)** miR-449a-5p and miR-98-5p expression were significantly and negatively correlated with KCNH2 expression; **(C–E)** miR-98-5p, miR-19b-3p, and miR-301a-3p expression were negatively correlated with KCNK15 expression. **(F)** There was no significant negative correlation between miR-138-5p and KCNK15 expression. **(G)** miR-30e-5p expression was positively correlated with KCNK15. **(H)** No significant negative correlation observed between miR-139-3p and SLC24A4 expression.

Pearson correlation analysis showed that miR-449a-5p (*r* = −0.89, *P* < 0.05) and miR-98-5p (*r* = −0.91, *P* < 0.05) expression were significantly and negatively correlated with KCNH2 expression (Figures 7A,B); miR-98-5p (*r* = −0.95, *P* < 0.05), miR-19b3p (*r* = −0.93, *P* < 0.01), and miR-301a-3p (*r* = −0.94, *P* < 0.01) were significantly and negatively correlated with KCNK15 expression (Figures 7C–E). However, we did not observe significant negative correlation between miR-138-5p (*r* = −0.36, *P* < 0.05)/miR30e-5p (*r*= 0.98, *P* < 0.01)/miR-139-3p (*r* = −0.74, *P*>0.05) expression and KCNK15/Slc24a4 expression (Figures 7F–H). miR-30e-5p expression was positively correlated with KCNK15 expression.

## Discussion

Currently, no treatment prevents the development of PTE, and symptom control is the primary goal of PTE management due to a lack of clinically applicable biomarkers and therapeutic targets (Saletti et al., 2019). In this study, considering the critical contribution of dysregulated potassium channels in PTE development (Wei et al., 2017), we used bioinformatics approaches including RNA sequencing, miRNA–mRNA network construction, and GO and KEGG enrichment analysis to identify significantly dysregulated miRNA–neuronal potassium channel mRNA interactions in an intracranial FeCl_2_ injection-induced PTE rat model. After further verification using qRT-PCR and correlation analysis, we identified five significantly and negatively correlated pairs of miRNA–potassium channel mRNA, including miR-449a-5p-KCNH2, miR-98-5p-KCNH2, miR-98-5p-KCNK15, miR-19b-3p-KCNK15, and miR-301a-3p-KCNK15. Among these interactions, KCNH2 and KCNK15 expressions were significantly upregulated, whereas miR-449a-5p, miR-98-5p, miR-98-5p, miR-19b-3p, and miR-301a-3p expressions were remarkably downregulated in the brain of PTE rats. Our findings provide new information for developing biomarkers and therapeutic targets for PTE prediction, prevention, and management.

In this study, we used FeCl_2_ to induce a PTE model in rats. Spontaneous seizures induced by TBI are required for a PTE model. Compared with physical induction (McIntosh et al., 1989; Dixon et al., 1991; Marmarou et al., 1994; Kharatishvili et al., 2006; Pitkänen and McIntosh, 2006; Mukherjee et al., 2013; Ostergard et al., 2016; Vink, 2018; Keith and Huang, 2019), chemical induction of PTE using FeCl_2_ has been extensively practiced and accepted, which is more similar to the natural physiological process of PTE formation. This is mainly because during the formation of PTE, hemosiderin plays an important role. Previous studies found that extravasation and dissolution of red blood cells and deposition of hemosiderin in neural networks (CNN) occur after TBI, which are typical symptoms of TBI and closely related to epilepsy (Yamamoto et al., 2002). As a result of trauma, subarachnoid hemorrhage and cerebral parenchymal hemorrhage often cause blood accumulated in the cortical tissue, which leads to a high risk of seizures. Animal experiments indicated that the epileptic effect of Fe ions is related to its redox reactions (Ueda et al., 2003). This may be due to the production of oxygen, light free radicals, and hydrogen peroxide as a result of oxidation of Fe iron. These substances can act on polyunsaturated fatty acids and cell membranes, causing subsequent transmission of dehydrogenation and peroxidation reactions. The cascade spread of such non-enzymatic lipid peroxidation reaction causes cell membrane rupture and changes in the microenvironment, leading to a high risk of PTE seizures (Wang et al., 2002; Meyerhoff et al., 2004). Therefore, chronic and spontaneous seizures could be caused by the deposition of iron-containing compounds on the cortex and edge structures of rat brains, suggesting FeCl_2_ could be effective in establishing the PTE model.

In this study, we successfully established the PTE rat model and identified four significantly upregulated potassium channel mRNAs in the PTE rat brain, including KCNH2, KCNK15, Slc24a4, and KCNN2. Except for KCNN2 without any matched miRNAs, we observed interactions between KCNH2/KCNK15/Slc24a4 and the differentially expressed miRNAs. KCNH2 encodes the α subunit for Kv11.1 potassium channel, and KCNH2 mutations cause type 2 long QT syndrome that typically presents with a seizure disorder or epilepsy (Omichi et al., 2010); however, whether the seizure or epilepsy is neurally mediated or due to a ventricular arrhythmia remains controversial (Johnson et al., 2009). A review of post-mortem records of sudden unexpected death in epilepsy cases has revealed the presence of KCNH2 mRNA alterations in these cases (Tu et al., 2011), suggesting that Kv11.1 dysregulation may be an independent factor in PTE pathogenesis. The α subunit is critical for the assembly of Kv11.1 tetramer. Downregulation of neuronal KCNH2 transcription leads to a significant decrease in potassium currents (Bertalovitz et al., 2018). The KCNH2-3.1 isoform transgenic mice exhibit abnormal neuronal firing patterns in the prefrontal cortex (Carr et al., 2016). Therefore, alterations in KCNH2 transcription may increase neuronal excitability, leading to the occurrence of seizures.

KCNK15 encodes a tandem-pore potassium channel TASK-5 that does not elicit ion currents by itself and requires other partners, such as TASK-3, to form functional channels (Karschin et al., 2001; Kim and Gnatenco, 2001). It has been reported that downregulation of KCNK15 transcription suppresses cell membrane depolarization to maintain potassium homeostasis (Dong et al., 2009), suggesting that KCNK15 overexpression might disrupt potassium homeostasis and lead to neuronal hyperexcitation.

Slc24a4 encodes a potassium-dependent sodium/calcium exchanger NCKX4 enriched in neurons (Li et al., 2002). In this study, the upregulation of Slc24a4 transcription in the PTE rat brain is possibly a response to hypoxia in neurons due to TBI (Neri et al., 2018). Hypoxia induces sodium/calcium influx and potassium efflux through inhibiting Na/K-ATPase, leading to neuronal hyperexcitation (Kiedrowski et al., 2004). Therefore, alterations in the sodium/calcium exchanger might also contribute to PTE development.

After constructing a miRNA–mRNA regulatory network, we identified seven miRNAs that were negatively correlated with KCNH2, KCNK15, or Slc24a4, including miR-138-5p, miR-19b-3p, miR-301a-3p, miR-30e-5p, miR-98-5p, miR-449a-5p, and miR-139-3p. Studies have shown decreased miR-449a-5p, miR-98-5p, and miR-138-5p induced by cerebral ischemia–reperfusion injury (Tang et al., 2016; Bernstein et al., 2019; Yu et al., 2019), decreased miR-30e-5p and miR-19b-3p induced by hypoxia (Mo et al., 2019; Liu et al., 2020), and decreased miR-30e-5p and miR-139-3p induced by inflammation (Budak et al., 2016; Cheng et al., 2019). Consistent with our results, researchers have observed decreased miR-138-5p and miR-30e-5p expression in a rat epilepsy model (Hu et al., 2012; Bot et al., 2013). Interestingly, contrasting results have been observed in different studies regarding the expression of miR-301a-3p. The miR-301a-3p serum level is reduced in patients with drug-resistant epilepsy and in rats with temporal lobe epilepsy (Hu et al., 2012; Wang et al., 2015), consistent with our results. In contrast, increased miR-301a-3p levels have been observed in the hippocampal and plasma of sudden unexpected death in temporal lobe epilepsy cases (De Matteis et al., 2018) and in the serum of patients with epilepsy (An et al., 2016). These discrepancies may arise from differences in the tissues and models.

After constructing a miRNA–mRNA network and analyzing the miRNA–mRNA correlation, we identified five significantly and negatively correlated miRNA–potassium channel mRNA pairs involved in PTE, including miR-449a-5p-KCNH2, miR-98-5p-KCNH2, miR-98-5p-KCNK15, miR-19b-3p-KCNK15, and miR-301a-3p-KCNK15. Although miR-138-5p, miR-30e-5p, and miR-139-3p expression were also downregulated in PTE rat brain, we did not observe significant negative correlations of them with any potassium channel mRNAs, suggesting that miR-138-5p, miR-30e-5p, and miR-139-3p might contribute to PTE by targeting other mRNAs.

To characterize the differentially expressed miRNAs in the PTE rat brain, we performed GO annotation and KEGG pathway enrichment analysis. We found that these miRNAs were closely associated with neuronal ion channels and neuroinflammation. TBI may induce neuroinflammation characterized by increased production and secretion of proinflammatory cytokines [such as tumor necrosis factor-α, interleukin (IL)-1β, and IL-6], reactive oxygen species, and neurotoxins (DiSabato et al., 2016). Elevated IL-1β levels in human cerebrospinal fluid and serum may increase the risk of PTE (Diamond et al., 2015a). Unlike IL-1β, tumor necrosis factor-α exerts dual effects on epilepsy (Balosso et al., 2005). In addition, the IL-6 levels are typically increased in the cerebrospinal fluid from TBI patients (Frugier et al., 2010), which is associated with the development of PTE (de Vries et al., 2016). In the results of KEGG analysis, we noticed that the differentially expressed miRNAs were enriched in “HIF-1signaling pathway” and “NF-kappa B signaling pathway,” suggesting that TBI-induced overproduction of proinflammatory cytokines may trigger the HIF-1 and NF- kappa B signaling pathways and contribute to the development of PTE through dysregulation of certain miRNAs.

As a single miRNA targets hundreds of mRNAs, miRNA dysregulation may lead to multiple and complicated biological consequences. Our results suggest that miR-301a-3p and miR-19b-3p downregulations are involved in PTE development; however, studies have shown that miR-301a-3p and miR-19b-3p downregulations alleviate neuroinflammation through inhibiting the activation of NF-kappa B signaling (Huang et al., 2013; Amjad et al., 2019). We speculate that TBI-induced miRNA dysregulation may alleviate neuroinflammation while increasing the excitability of neuronal ion channels through different mRNA targets. On the other hand, downregulations of miR-449a-5p, miR-98-5p, and miR-301a-3p expressions observed in our PTE model have been shown to promote neuroinflammation. For example, miR-449a-5p is commonly downregulated in ischemic brain injury and atherosclerosis, whereas miR-449a-5p overexpression alleviates inflammation through NF-kappa B signaling (Jiang et al., 2019; Yu et al., 2019). IL-6 is a direct target of miR-98-5p, and loss of miR-98-5p exacerbates inflammation through upregulating IL-6 expression (Ji et al., 2016). In addition, miR-301a-3p upregulation exerts neuroprotective effects through target mRNAs (Chen et al., 2016).

Our study has some limitations, including a lack of verification regarding the physical bindings between miRNAs and mRNAs and focusing only on an animal model. These limitations will be addressed in future studies.

In conclusion, in this study, we demonstrated that five miRNA–potassium channel mRNA pairs, including miR-449a-5p-KCNH2, miR-98-5p-KCNH2, miR-98-5p-KCNK15, miR-19b-3p-KCNK15, and miR-301a-3p-KCNK15, were significantly differentially expressed in the brain of PTE rats. Our results suggest that the alterations in these miRNAs and miRNA-regulated potassium channel mRNA expression are involved in the development of PTE, providing potential diagnostic biomarkers and therapeutic targets for PTE management.

## Data Availability Statement

The datasets for this study are available this in the NCBI website, accessible with the following link, the Bioproject ID is PRJNA667324 (http://www.ncbi.nlm.nih.gov/bioproject/667324).

## Ethics Statement

The animal study was reviewed and approved by Animal Research Ethics Committee of the Institute of Evidence Science, China University of Political Science and Law.

## Author Contributions

TY and QL conceived and designed the study. ZL and YM analyzed the data and wrote the initial draft of the manuscript. FZ, JZ, XJ, and HT conducted the experiments and collected the data. XW contributed to refining the ideas. All authors were involved in revising the manuscript.

## Conflict of Interest

The authors declare that the research was conducted in the absence of any commercial or financial relationships that could be construed as a potential conflict of interest.
