# Supplementary material for: Identification of MicroRNA–Potassium Channel Messenger RNA Interactions in the Brain of Rats With Post-traumatic Epilepsy
Source: Front Mol Neurosci. 2021 Feb 1;13:610090. doi: 10.3389/fnmol.2020.610090 (PMC7882489; doi:10.3389/fnmol.2020.610090)
Supplement: Supplementary file 1 [file Data_Sheet_1.docx]

Supplementary Material

# Supplementary Figures and Tables

## Supplementary Figures


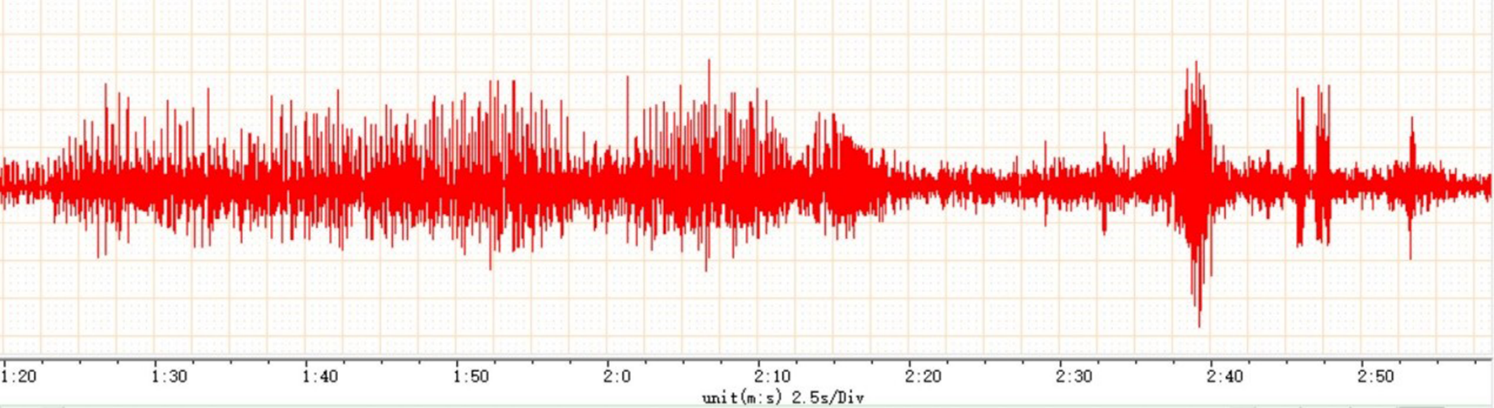


**Supplementary Figure 1.**Representative images of EEG records of PTE seizures rats for 1min and 30 s with abnormal discharge observed on day 30.


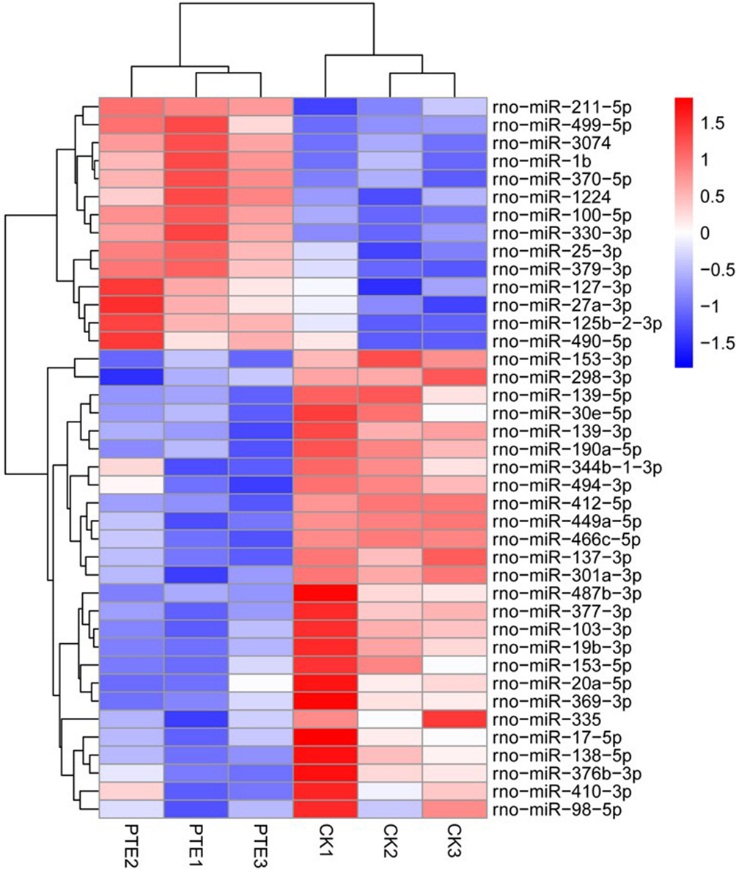


**Supplementary Figure 2.**Differentially expressed miRNA. High-throughput sequencing was performed to identify differentially expressed microRNAs between PTE and sham rats.

## Supplementary Tables

**Supplementary Table 1.**Racine’s seizures scores for each experimental animal

| Time | Code of sample | | | | | | | | |
| --- | --- | --- | --- | --- | --- | --- | --- | --- | --- |
|  | 1# | 2# | 3# | 4# | 5# | 6# | 7# | 8# | 9# |
| Prior to modelling | 0 | 0 | 0 | 0 | 0 | 0 | 0 | 0 | 0 |
| 60 min Post-modelling | 5 | 5 | 5 | 4 | 4 | 5 | 5 | 5 | 5 |
| day 1 | 4 | 4 | 5 | 5 | 4 | 4 | 4 | 5 | 5 |
| day 2 | 5 | 4 | 2 | 5 | 5 | 5 | 5 | 4 | 4 |
| day 3 | 4 | 5 | 4 | 3 | 3 | 4 | 5 | 4 | 4 |
| day 4 | 3 | 5 | 4 | 5 | 5 | 5 | 5 | 4 | 3 |
| day 5 | 5 | 4 | 2 | 4 | 4 | 5 | 4 | 5 | 4 |
| day 6 | 5 | 5 | 5 | 5 | 5 | 3 | 5 | 5 | 5 |
| day 7 | 4 | 4 | 5 | 5 | 5 | 5 | 2 | 6 | 4 |
| day 8 | 5 | 5 | 1 | 5 | 3 | 3 | 5 | - | 5 |
| day 9 | 5 | 4 | 5 | 5 | 4 | 5 | 4 | - | 5 |
| day 10 | 4 | 5 | 4 | 3 | 5 | 4 | 5 | - | 5 |
| day 11 | 5 | 5 | 5 | 5 | 4 | 5 | 5 | - | 5 |
| day 12 | 4 | 4 | 5 | 4 | 4 | 3 | 4 | - | 5 |
| day 13 | 5 | 5 | 5 | 5 | 5 | 5 | 5 | - | 4 |
| day 14 | 5 | 3 | 4 | 4 | 5 | 5 | 4 | - | 5 |
| day 15 | 4 | 5 | 5 | 5 | 3 | 4 | 5 | - | 4 |
| day 16 | 5 | 4 | 6 | 5 | 5 | 5 | 5 | - | 5 |
| day 17 | 5 | 5 | - | 1 | 4 | 1 | 4 | - | 5 |
| day 18 | 4 | 5 | - | 5 | 5 | 5 | 5 | - | 5 |
| day 19 | 5 | 5 | - | 5 | 5 | 3 | 5 | - | 4 |
| day 20 | 4 | 4 | - | 5 | 4 | 5 | 4 | - | 5 |
| day 21 | 5 | 3 | - | 4 | 5 | 3 | 5 | - | 6 |
| day 22 | 1 | 5 | - | 5 | 4 | 5 | 4 | - | - |
| day 23 | 5 | 5 | - | 5 | 5 | 5 | 5 | - | - |
| day 24 | 4 | 4 | - | 2 | 4 | 4 | 4 | - | - |
| day 25 | 5 | 5 | - | 5 | 3 | 5 | 5 | - | - |
| day 26 | 3 | 5 | - | 5 | 4 | 5 | 5 | - | - |
| day 27 | 5 | 3 | - | 4 | 1 | 5 | 1 | - | - |
| day 28 | 2 | 5 | - | 4 | 3 | 4 | 5 | - | - |
| day 29 | 5 | 2 | - | 5 | 4 | 5 | 4 | - | - |
| day 30 | 4 | 5 | - | 5 | 5 | 5 | 5 | - | - |
